# The Present Status of the Medical Colleges of Georgia

**Published:** 1892-08

**Authors:** 


### *EDITORIAL.*

The office of THE JOURNAL is in rooms 43 and 44 Old Capitol Building.
Address communications relating to the Editorial Department to DR. L. B. GRANDY, Atlanta, Ga., Box 431.
Business communications should be addressed to DR. M. B. HUTCHINS, Atlanta, Ga.
All remittances should be made payable to "ATLANTA MEDICAL AND SURGICAL JOURNAL."
Articles for publication should reach this office not later than the 15th instant.
The editors are not responsible for views expressed by contributors.

### THE PRESENT STATUS OF THE MEDICAL COLLEGES OF GEORGIA.

We have before us the latest reports of the examining boards of Alabama and Virginia, which are anything but flattering to some of our Georgia medical colleges. No better argument in favor of examining boards could be offered than the simple presentation of these reports, part of which we append. We give only the table for Southern colleges.

MEDICAL COLLEGES.

| SOUTHERN. | Applied. | Granted. | Rejected. |
|---|---|---|---|
| University of Nashville | 14 | 13 | 1 |
| Vanderbilt University | 92 | 88 | 4 |
| Meharry Medical College (Nashville) | 5 | 2 | 3 |
| Memphis Hospital Medical College | 10 | 8 | 2 |
| University of Louisville | 18 | 14 | 4 |
| Kentucky School of Medicine (Louisville) | 16 | 15 | 1 |
| Louisville Medical College | 26 | 20 | 6 |
| Louisville Hospital Medical College | 4 | 4 | 0 |
| Central University (Nashville) | 1 | 0 | 1 |
| Transylvania University (Lexington) | 6 | 5 | 1 |
| Georgia Medical College (Augusta) | 8 | 6 | 2 |
| Atlanta Medical College | 42 | 28 | 14 |
| Southern Medical College (Atlanta) | 20 | 17 | 3 |
| Georgia Eclectic Coll. Reform (Atlanta) | 10 | 6 | 4 |
| Savannah Medical College | 1 | 1 | 0 |
| Alabama Medical College (Mobile) | 122 | 115 | 7 |
| University of Louisiana—Tulane (N. O.) | 25 | 25 | 0 |
| Virginia Medical College (Richmond) | 7 | 7 | 0 |
| University of Virginia (Charlottesville) | 13 | 13 | 0 |
| University of Maryland (Baltimore) | 10 | 9 | 1 |
| Baltimore Medical College | 1 | 0 | 1 |
| Physicians and Surgeons, of Baltimore | 13 | 12 | 1 |
| Washington University | 1 | 1 | 0 |
| Howard University (Washington) | 6 | 2 | 4 |
| University of Tennessee (Nashville) | 30 | 25 | 5 |
| South Carolina Medical College (Charleston) | 6 | 5 | 1 |
| Total | 507 | 441 | 66 |

RECAPITULATION. (All Colleges.)

| | |
|---|---|
| Whole number applied | 647 |
| Whole number granted | 559 |
| Whole number rejected | 88 |
| Percentage of rejections | 13.50 |
| Percentage of rejections of graduates Western Medical Colleges | 19.40 |
| Percentage of rejections of graduates Southern Medical Colleges | 13.01 |
| Percentage of rejections of graduates Northern Medical Colleges | 9.45 |
| Percentage of rejections of non-graduates | 28.57 |
| Percentage of rejections of graduates (College unknown) | 33.03 |
| Percentage of rejections of foreign graduates | 0.00 |

In Virginia one graduate from the Atlanta Medical College and two from the Southern Medical College have applied for license to practice medicine and all been rejected. In Alabama the results have been more favorable to the Georgia colleges. It will be seen that the Southern Medical College has a record of twenty applications and three rejections. Augusta comes second with eight applications and two rejections, while the Atlanta Medical College presents a record of forty-two applications and fourteen rejections. Percentage of rejections of graduates of Southern Medical College, fifteen; of Medical Department of the University of Georgia, twenty-five; of Atlanta Medical College thirty-three and one third.

We make no comparisons, but only give figures for what they are worth. It is sadly to the discredit of the Georgia colleges that fifteen, twenty-five and thirty-three per cent. of the men whom they present with diplomas should be declared by the Board of one State to be unfit to practice medicine. The public has a deep and vital interest in this matter, and as a progressive and independent journal, the friend of all the colleges, but the organ of none, we are determined to keep before the people the necessity of a higher medical education.

But just so long as the success of a medical college is measured by the size of its classes and the amount of money received and disbursed, just so long will this state of affairs continue to exist.

Nor should this condition of things be lightly considered by the citizens of Georgia who are now virtually at the mercy of the medical colleges. Nothing is required of any one who wishes to practice medicine in this State, except the registration of a diploma from a regularly chartered medical college, no matter what the professional status of that college may be. The laity cannot properly estimate the capabilities of medical men and must rely on the State and colleges for protection. And it is the sacred duty of the State to provide such laws as will protect its citizens and make it impossible for a medical college to foist into a community an ignoramus who is far more dangerous than an epidemic. Think of having to confide one's wife or one's sister requiring the services of a skilled obstetrician or surgeon into the hands of one of these impostors who have not the slightest knowledge of anatomy or the principles of obstetrics or surgery! And yet this is done almost daily. We do not bring the matter home to ourselves or we would not be so derelict in our duties as medical men.

Georgia is now the dumping ground for the rag-tag and bob-tail of the profession who are rejected by the examining boards of the surrounding States. Can Georgia, the Empire State of the South, longer afford to have the finger of scorn pointed at her as the hot bed of quackery and the foster mother of medical impostors?

We hope to see our colleges aroused to a sense of their responsibilities in these matters, for all that is necessary is to get the people awakened to the dangers of the situation and they will demand of their representatives the passage of better laws.

